# Comparison of the Efficacy of Pirfenidone and Nintedanib in the Treatment of Patients with Idiopathic Pulmonary Fibrosis—A Single-Center Experience

**DOI:** 10.3390/medicina62010229

**Published:** 2026-01-22

**Authors:** Nikola Trboljevac, Sanja Dimic-Janjic, Milica Kontic, Maja Omcikus, Branislav Ilic, Filip Markovic, Anka Postic, Lidija Isovic, Mihailo Stjepanovic, Dragana Nenezic

**Affiliations:** 1 Clinic for Pulmonology, University Clinical Center of Serbia, 11000 Belgrade, Serbia; 2 Faculty of Medicine, University of Belgrade, 11000 Belgrade, Serbia; 3 Clinic for Otorhinolaryngology, University Clinical Center of Serbia, 11000 Belgrade, Serbia

**Keywords:** idiopathic pulmonary fibrosis, progression, real-world cohort, usual interstitial pneumonia, antifibrotic treatment comparison

## Abstract

*Background and Objectives:* Idiopathic pulmonary fibrosis (IPF) is a progressive, unpredictable, fatal interstitial lung disease. Antifibrotic therapy with pirfenidone or nintedanib slows functional decline, yet comparative real-world evidence remains limited. *Materials and Methods:* This retrospective, single-center, comparative cohort study included 76 IPF patients treated at the Clinic for Pulmonology at the University Clinical Center of Serbia (February 2019–February 2025). Diagnosis of IPF was made according to the guidelines of the American Thoracic Society and the European Respiratory Society. Demographic features, comorbidities, forced vital capacity (FVC), diffusion capacity for carbon monoxide (DLCO), high-resolution computerized tomography (HRCT) patterns, 6-min walk test distance (6MWTD), echocardiography, and survival outcomes were analyzed. Disease progression was defined as a ≥10% decline in FVC and/or DLCO after 12 months. *Results:* Of the 76 patients, 31 received nintedanib and 45 pirfenidone. Baseline characteristics, comorbidities, and HRCT patterns were comparable between groups. Mean annual decline in FVC was −1.74% with pirfenidone and −2.38% with nintedanib, without a statistical difference. DLCO declined by −4.25% and −6.29%, respectively, with similar downward trends over time in both groups. Progression was recorded in 35 (46.1%) patients, of whom 18 (58.06%) were in the nintedanib group and 17 (37.77%) in the pirfenidone group, with no difference between therapies (*p* = 0.81). Definite and probable usual interstitial pneumonia (UIP) were evenly represented on HRCT, although progression correlated significantly with the probable UIP pattern (*p* = 0.006). 6MWTD decreased in both groups over 12 months, again without treatment-related differences (*p* = 0.566). During up to 6 years of follow-up, overall survival was 4.18 years, with no significant difference between the nintedanib (4.55 years) and pirfenidone (3.81 years) groups (*p* = 0.159). No association was found between disease stage (FVC or DLCO) and progression. *Conclusions:* This study demonstrates that pirfenidone and nintedanib are equally effective in the management of IPF in real-world settings. The absence of significant differences in functional decline, progression rates, and survival indicates that treatment choices should be guided by individual clinical profiles rather than efficacy alone, reinforcing antifibrotic therapy as the primary approach to alter the course of IPF. Importantly, disease progression was strongly associated with a probable UIP pattern on HRCT, supporting current guidelines suggesting that probable UIP has a natural history and prognosis similar to those of definite UIP.

## 1. Introduction

Idiopathic pulmonary fibrosis (IPF) is a chronic, fibrosing interstitial lung disease characterized by an unpredictable clinical course and limited survival. The incidence and prevalence of IPF vary by country or region, case definition, and the demographic features of the studied population. Adjusted incidence estimates (per 10,000 population) range from 0.35 to 1.30 in Asia-Pacific countries, 0.09 to 0.49 in Europe, and 0.75 to 0.93 in North America, with the highest incidence reported in South Korea, Canada, and the United States. Adjusted prevalence estimates range from 0.57 to 4.51 in the Asia-Pacific region, 0.33 to 2.51 in Europe, and 2.40 to 2.98 in North America, per 10,000 population. Both the highest incidence and prevalence are observed in South Korea [1]. From a pathophysiological perspective, IPF is no longer seen primarily as an inflammatory condition but as a disorder of abnormal wound healing driven by repetitive epithelial injury, fibroblast activation, and excessive extracellular matrix deposition [2]. Dysregulated signaling pathways—including transforming growth factor-β (TGF-β), platelet-derived growth factor (PDGF), fibroblast growth factor (FGF), and vascular endothelial growth factor (VEGF)—play key roles in initiating and sustaining fibrotic remodeling [3]. Once established, fibrosis becomes self-perpetuating and largely irreversible, highlighting the importance of therapies aimed at slowing, rather than reversing, disease progression. A key principle of the diagnostic approach for IPF is the systematic exclusion of other interstitial lung diseases that may mimic its clinical and radiologic presentation, enabling an accurate multidisciplinary diagnosis and the selection of an optimal treatment strategy. Pulmonary function testing reflects the progressive nature of the disease and remains essential for risk assessment and long-term monitoring. Radiologic assessment, especially high-resolution computed tomography (HRCT), is central to diagnosis, with identification of a usual interstitial pneumonia (UIP) pattern being crucial. In cases with indeterminate HRCT findings or conflicting clinical and radiologic features, surgical lung biopsy or transbronchial lung cryobiopsy may be considered. Nevertheless, a multidisciplinary discussion that integrates clinical, radiologic, and, when available, histopathologic data remains the gold standard for diagnosis. Pirfenidone and nintedanib are currently the only approved disease-modifying agents for IPF, targeting the underlying pathophysiological processes. Their antifibrotic effects have proven clinically relevant in altering the natural course of this severe disease. Despite the lack of direct comparative randomized controlled trials, available evidence from narrative reviews and real-world practice suggests that pirfenidone and nintedanib are comparable in their effectiveness at slowing disease progression, with no agent showing clear superiority [4]. Differences in comorbidities, disease stage, tolerability, adherence, and patient-specific risk factors can significantly influence outcomes in routine clinical settings, beyond efficacy alone [5]. Therefore, real-world cohort studies, such as this one, offer valuable additional insights by reflecting everyday clinical decision-making and long-term outcomes. The results of this study show that pirfenidone and nintedanib are similarly effective in decelerating functional decline and disease progression. Additionally, the progression rates and survival data are consistent with previously reported registry findings, supporting the external validity of the cohort. Overall, these findings support the current treatment approach, in which antifibrotic therapy remains the cornerstone of IPF management, and treatment choices should be tailored to individual patient characteristics, comorbidities, safety profiles, and preferences.

## 2. Materials and Methods

This study was designed as a retrospective, observational, comparative noninterventional study that included 76 patients treated at the Clinic for Pulmonology at the University Clinical Center of Serbia (UCCS) in Belgrade, Serbia, from February 2019 to February 2025. The Clinic’s internal medical board and Ethical board approved the study. Diagnosis of IPF was made according to the guidelines of the American Thoracic Society and the European Respiratory Society [6], and a multidisciplinary team consisting of a pulmonologist, radiologist, and rheumatologist, with thoracic surgeons and pathologists involved as needed, confirmed the IPF diagnosis. The institutional Fibrosis Expert Board recommended the type and dosage of antifibrotic therapy, as recommended by the Serbian drug agency (ALIMS) and the manufacturer.

The study included all eligible patients treated with pirfenidone or nintedanib at our center during the predefined study period. Therefore, the sample size reflects real-world clinical practice and was determined by patient availability rather than statistical power considerations.

Inclusion criteria were: IPF diagnosis confirmed by the institutional Fibrosis Expert Board based on the available data presentation, regardless of age and functional capacity, and treatment-naïve.

Exclusion criteria were: the exclusion of other interstitial lung diseases that may mimic IPF, lung transplantation, and drug discontinuation before the end of the 6-month treatment period.

Demographic data, comorbidities, pulmonary function parameters, echocardiographic findings, and HRCT patterns were collected from electronic medical records (Heliant system). Functional assessments followed standards set by the American Thoracic Society (ATS) and European Respiratory Society (ERS) [7,8]. Spirometry and diffusing capacity for carbon monoxide were measured using the JAEGER^®^ MasterScreen Pneumo system (Vyaire Medical GmbH, Hochberg, Germany). HRCT scans were acquired with a 128-slice Siemens Somatom Definition Edge scanner (Siemens Healthineers, Forchheim, Germany). HRCT was performed using a standard protocol, with images acquired at full inspiration in the supine position, craniocaudal direction, in the non-contrast phase, and reconstructed with a lung kernel (No. 46, 0.6 mm). The six-minute walk test (6MWT) was performed according to ATS guidelines [9]. Survival outcomes were evaluated through medical record review.

The primary objective of the study was to investigate which oral antifibrotic drug is superior in preventing functional disease progression in patients with IPF, by comparing functional deterioration between patients treated with pirfenidone and those treated with nintedanib. Disease progression was defined as ≥10% decline in FVC and/or DLCO after 12 months. Pulmonary function tests, including FVC (% predicted) and DLCO (% predicted), were measured at baseline, after 6 months, and one year of treatment.

In a secondary objective manner, we tested whether there were differences between groups in age, BMI, smoking status, comorbidities, UIP pattern, 6MWTD, and survival. Also, we tested the correlation between progression and elevated RVSP values, UIP pattern, DLCO, and FVC stage.

Disease severity at baseline was evaluated using available clinical and functional parameters at the time of cohort inclusion. Pulmonary function tests, including FVC (% predicted) and DLCO (% predicted), were used as indicators of disease severity. Given the retrospective design of the study, no composite severity score was applied; however, baseline functional parameters did not differ substantially between treatment groups, supporting their comparability.

Missing data were infrequent and were handled using complete-case analysis; no data imputation was performed. Analyses were based on available data at each time point. Given the retrospective observational design, no formal adjustment for confounders was performed; however, baseline demographic and functional characteristics were assessed to ensure comparability between treatment groups. All patients diagnosed with IPF during the observed period were included, without selection bias.

### Statistical Analysis

Statistical analysis was performed using SPSS (SPSS for Windows, release 26.0, SPSS, Chicago, IL, USA). Descriptive statistical methods, methods for testing statistical hypotheses, and methods for examining associations were used for the analysis of primary data. The following descriptive methods were used: absolute and relative numbers; measures of central tendency and measures of variability. Data distribution was assessed using histograms and the Shapiro–Wilk test. Normally distributed variables are presented as mean ± standard deviation. To compare statistically significant differences between the examined groups, the χ^2^ test (or Fisher’s exact test) was used for nominal data. Student’s *t*-test (or the Mann–Whitney test) was used to analyze differences between two groups, depending on the data distribution, and repeated-measures ANOVA. Time points selected for repeated measurements of pulmonary function tests are baseline (when therapy was initiated), 6 months, and one year of therapy. Correlation was tested using Spearman’s correlation. Survival was estimated using the Kaplan–Meier method and compared between groups using the log-rank (Mantel–Cox) test. Overall survival was defined as time to death from any cause; patients who were alive at the end of follow-up or lost to follow-up were censored at their last known contact. Statistical hypotheses were tested at a statistical significance level of alpha 0.05.

## 3. Results

Baseline characteristics of the examined population at the start of the study are similar, with no notable differences between treatment groups; a complete overview is provided in Table 1. A total of 76 patients were included in the analysis, of whom 31 (40.8%) were treated with nintedanib and 45 (59.2%) with pirfenidone. Males were predominant (54; 71.1%), whereas females accounted for 22 (28.9%) of all included patients. Mean BMI was in the overweight range. In the examined population, a considerably higher proportion of patients (66, or 73.7%) smoked cigarettes. There is no statistically significant difference between the two groups in age, BMI (*p* = 0.531 and 0.854, respectively), or smoking status (*p* = 0.287).

Only 5 of the 76 patients had no comorbidities. Of all comorbidities, the highest prevalence in both therapeutic groups was arterial hypertension (HTN) (77.46%), diabetes mellitus (28.16%), COPD (16.9%), and PH (14.08%). The most common finding was 2 comorbidities per affected patient (29; 38.2%). The number of comorbidities was similarly presented across the therapeutic groups (*p* = 0.643), with no difference between groups in the most common comorbidities (arterial hypertension, *p* = 0.786; diabetes, *p* = 0.32; PH, *p* = 0.514; COPD, *p* = 1.0).

Progression was recorded in 35 (46.1%) patients, with 18 (58.06%) in the nintedanib group and 17 (37.77%) in the pirfenidone group, with no statistically significant difference between the groups (*p* = 0.81).

The dominant HRCT pattern was definite UIP (39), but probable UIP (37) was equally represented. HRCT patterns in relation to treatment are not statistically significant (*p* = 0.174). In patients with progression, the main HRCT finding is probable UIP (23), while typical UIP is observed in 12 subjects. There is a statistically significant correlation between progression and a probable UIP pattern (*p* = 0.006).

Functional evaluation was conducted at therapy initiation, at 6 months, and at 1 year. The one-year decline in FVC values was approximately 2%, and DLCO about 5% in the studied cohort. There is no statistically significant difference in the means of FVC in relation to treatment at all three measurements (*p* = 0.554, *p* = 0.962, *p* = 0.489), and there was no effect of treatment on this change (*p* = 0.383). DLCO testing showed no differences in relation to three measurements and treatment (*p* = 0.672, *p* = 0.949, *p* = 0.875), (*p* = 0.667), respectively. FVC change is not statistically significant at certain time intervals within the same group. However, there was a statistically significant difference between measurements at certain time intervals within the same group: in the nintedanib group, between the first and second, and first and third measurements (*p* = 0.017, *p* = 0.004), respectively. In the pirfenidone group, similar differences were observed between the first and second, and the first and third measurement (*p* = 0.02, *p* = 0.008), respectively. The trend of DLCO decline is observed across all measurements and in both groups. Mean ± SD of FVC and DLCO was shown in Table 2 at specific time points.

Over time, the distance decreases progressively in both groups, with a slightly greater decrease in the pirfenidone group, as shown in Table 3 (mean ± SD). There is no statistically significant difference in the change of mean ± SD walking distance related to treatment at any of the time point measurements (*p* = 0.977, *p* = 0.945, *p* = 0.402), nor in the difference in efficacy in the context of treatment and time (*p* = 0.566, *p* = 0.344), respectively. The only statistically significant difference occurs between the second and third measurements in patients treated with pirfenidone, but there is no overall treatment effect on this difference (*p* = 0.032).

As part of the IPF evaluation, patients also underwent an echocardiogram (ECHO) to assess right ventricular systolic pressure (RVSP) and ejection fraction (EF). The mean RVSP value in the nintedanib group was 38.5 mmHg, while in the pirfenidone group it was 41.71 mmHg. EF was preserved in both groups: nintedanib 54.61%, pirfenidone 57%. Elevated RVSP values (>40 mmHg) were observed in 30 patients. In 13 of these (43%), progression was accompanied by increased values. There is no statistically significant association between progression and elevated RVSP values in our cohort (*p* = 0.455).

The stage of pulmonary fibrosis was classified based on DLCO and FVC values, as presented in Table 4 [10]. DLCO stages were defined as: mild (>55% predicted), moderate (35–55%), severe (<35%). Most patients (48) were in DLCO stage 3, including 31 treated with pirfenidone and 17 with nintedanib. FVC stages were defined as: mild (>80%), moderate (50–79%), severe (<50%). The majority of patients (40) were in FVC stage 1, with 23 treated with pirfenidone and 17 with nintedanib. There were no statistically significant differences in therapy by DLCO or FVC stage (*p* = 0.294 and *p* = 0.855, respectively). Additionally, no association was found between DLCO or FVC stages and disease progression (*p* = 0.34 and *p* = 0.887).

Survival was defined as the time from cohort inclusion (initiation of antifibrotic therapy) to death from any cause. Patients who were alive at the end of follow-up or lost to follow-up were treated as censored observations. Follow-up extended through 2025, with a maximum observation period of 6 years.

During follow-up, 27 patients (35.5%) died, while 49 patients (64.5%) were censored. Two patients were followed for the full 6-year period, 16 patients for 1 year, and the highest number of censored observations occurred at 3 years (*n* = 25).

**Figure 1:**
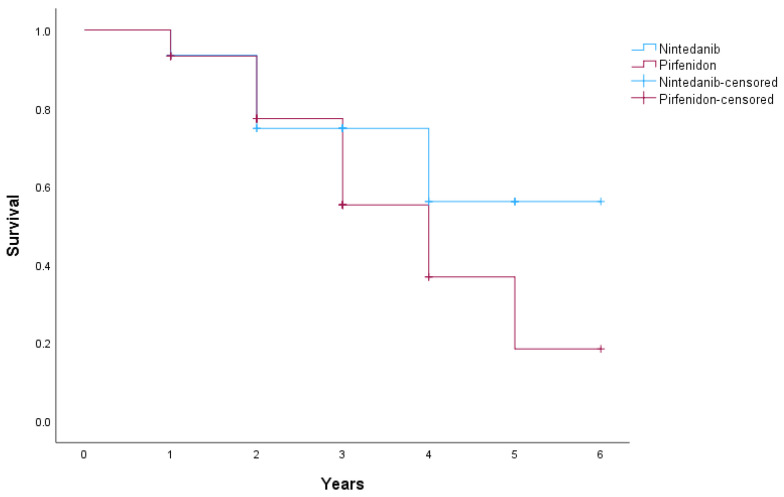
Survival is expressed in years relative to treatment.

In the nintedanib group (*n* = 31), 9 patients (29%) experienced the event (death), while 22 patients (71%) were censored. In the pirfenidone group (*n* = 45), 18 patients (40%) died, and 27 patients (60%) were censored. The mean estimated survival time was 4.55 years for patients treated with nintedanib and 3.81 years for those treated with pirfenidone. The overall mean survival time for patients receiving antifibrotic therapy was 4.18 years. Analysis demonstrated no statistically significant difference in overall survival between treatment groups (*p* = 0.159). Kaplan–Meier survival curves stratified by antifibrotic treatment are shown in Figure 1.

## 4. Discussion

In this real-world cohort of patients with IPF, treatment with pirfenidone and nintedanib resulted in comparable clinical outcomes. No statistically significant differences were observed between treatment groups in terms of lung function decline or overall survival. Declines in FVC were modest over one year, while DLCO showed a more pronounced reduction, supporting its role as a sensitive marker of disease progression. Overall, our findings reinforce the effectiveness of antifibrotic therapy in slowing functional deterioration in IPF and highlight the importance of individualized treatment selection.

Following these findings, treatment allocation in our cohort reflected real-world clinical decision-making. Our fibrosis expert board decided that a slightly larger proportion of patients would be treated with pirfenidone. When making this decision, multiple patient-related factors were considered, including comorbidities, clinical characteristics, patient preferences, concomitant therapies, and drug availability. Guided by the available evidence, therapeutic decisions were made on a case-by-case basis. Differences in safety profiles—such as a higher incidence of gastrointestinal adverse events and liver enzyme abnormalities with nintedanib and increased photosensitivity with pirfenidone—influenced treatment selection [11]. Importantly, clinical characteristics such as bleeding risk, as well as comorbidities and concomitant medications, can significantly affect drug tolerability and overall risk profile [12]. Furthermore, patient-reported experiences and satisfaction highlight the importance of shared decision-making in optimizing treatment adherence and quality of life [13].

To further contextualize these findings, we examined changes in pulmonary function over time, focusing on FVC and DLCO as key markers of disease progression. Decline in FVC over one year was modest, −2.38% ± 14.56% in the nintedanib group and −1.74% ± 11.22% in the pirfenidone group—aligning with prior real-world data on patients on antifibrotic treatments [14]. The extent of FVC decline in our cohort matches that observed in the ASCEND and INPULSIS trials, supporting the external validity of our real-world results [15,16]. Conversely, DLCO experienced a more notable decline over time, with an overall one-year reduction of −5.02% ± 10.11%, observed across all measurements and both treatment groups. Although the decline was numerically greater in the nintedanib group (−6.29% ± 11.75% vs. −4.25% ± 9.04%), this difference should be interpreted with caution.

The less prominent decline in FVC compared with DLCO suggests that volume-based parameters may be less sensitive to subtle functional changes in certain patients. This observation is supported by evidence indicating that disease progression in IPF is heterogeneous and that longitudinal changes in FVC are variable and may poorly predict individual trajectories [17]. While a decline in FVC of ≥10% within 6 months is a recognized predictor of mortality [18], DLCO values below 35% of predicted and longitudinal declines in DLCO have been shown to be strong prognostic markers, even in patients with relatively stable FVC [19]. Although FVC and DLCO are physiologically correlated, declines in DLCO may occur without significant changes in FVC, potentially reflecting microvascular injury and alveolar–capillary membrane dysfunction that are not captured by spirometric measurements alone [20]. This provides a theoretical rationale for using DLCO as a complementary marker of disease progression in our study. In contrast to our findings, in which the effectiveness of both drugs in slowing functional decline is comparable, an Italian real-world study reported a more pronounced decline in DLCO after 12 months in patients treated with pirfenidone, a finding that is hypothetically attributed to nintedanib’s antiangiogenic properties [21].

The six-minute walk test distance (6MWTD) is the most commonly used method for assessing exercise capacity in patients with IPF [22]. A change in walking distance of about 30 m is considered clinically significant in patients with IPF, and lower recorded oxygen saturation levels during the test are linked to a poorer prognosis [23,24]. Measuring 6MWD as a secondary endpoint in this study follows the approach used in nearly all phase 3 trials evaluating the effectiveness of oral antifibrotics [15,16]. The distance decreases over time for both groups, with a slightly greater decline in the pirfenidone group. A particularly notable drop occurs between the 6-month period and the 1-year post-treatment period. Baseline characteristics were comparable between treatment groups, reducing the likelihood that this finding reflects baseline imbalance. Importantly, assessment of adverse events, clinical status, and other factors influencing exercise capacity was not within the scope of the present study. Given the multifactorial nature of the six-minute walk test, the observed trend may reflect unmeasured influences rather than differences in antifibrotic efficacy. Overall, these findings are consistent with the absence of a clinically meaningful difference between pirfenidone and nintedanib in preserving exercise capacity over time.

The most common pulmonary comorbidities are COPD, with a prevalence of 37.34%, and lung cancer, at 3.34%, while the most common extrapulmonary comorbidities include GERD (70.83%), dyslipidemia (62.93%), and arterial hypertension (59.04%) [25]. In our study, most patients had two comorbidities, specifically HTN and DM. Lung cancer accounted for 6.57% of cases, making it relatively rare compared to the literature. A meta-analysis of 11 studies showed its prevalence ranged from 6.77% to 31.31%, with significant heterogeneity among studies [26]. While comorbidities such as hypertension, diabetes mellitus, and lung cancer may affect overall prognosis and functional status in patients with IPF, assessing their impact on treatment response and disease progression was not the objective of this study. Therefore, no formal analysis was conducted to evaluate the influence of these comorbidities on lung function decline, exercise capacity, or survival outcomes. Given the retrospective nature of the study and the limited detailed data on comorbidities, the influence of these conditions on treatment outcomes should be interpreted with caution. Future prospective studies with comprehensive comorbidity assessments are needed to better understand their role in modifying responses to antifibrotic treatment.

In our cohort, a probable UIP pattern was associated with disease progression. These findings are in contrast to real-world data, which suggest that definite UIP is more commonly observed among progressors [27]. Probable UIP likely represents an earlier or more dynamic stage within the UIP spectrum, characterized by less established fibrosis and greater residual functional lung parenchyma. Consequently, these patients may be more susceptible to measurable functional decline over time. In contrast, patients with a definite UIP pattern have more advanced and fixed fibrotic changes, which may limit the detection of further progression due to severely impaired lung function parameters. Importantly, current guidelines and previous studies suggest that probable UIP shares a similar natural history and prognosis with definite UIP, supporting the biological plausibility of our findings [6].

The median survival in patients with IPF is estimated at 3 to 5 years from diagnosis. In adults in the USA older than 65 years who had IPF between 2001 and 2011, the median survival was 3.8 years [28]. In a Canadian study conducted from 2007 to 2011, the risk of death within four years was 41% [29]. An Italian real-world study conducted between 2011 and 2019 reported a median survival of 1224 days, which is comparable to survival outcomes reported in other real-world cohorts and consistent with the follow-up observed in our study [21]. Several studies have shown improved survival over the past decade, which may be attributed to earlier diagnosis, reduced use of immunosuppressive drugs, and the introduction of more effective therapies. A more recent study showed a decline in standardized mortality rates for interstitial lung disease (ILD) across several European countries, suggesting that advances in diagnosis and treatment may have contributed to this improvement [30]. According to data by Mura et al., during a 3-year follow-up of 70 patients, 32 died (which is about 46% of the participants). In our cohort, overall survival at a maximum follow-up of 6 years did not differ significantly between patients treated with pirfenidone and those treated with nintedanib. Although the mean estimated survival time was numerically longer in the nintedanib group, this difference was not statistically significant and should therefore be interpreted with caution. The observed survival outcomes, with an overall mean survival of approximately 4.2 years, are consistent with previously reported real-world data, supporting the notion that antifibrotic therapy favorably modifies the natural course of IPF without conferring a clear survival advantage for any drug. As expected in observational cohorts, a substantial proportion of patients were censored, and survival estimates at later time points should be interpreted cautiously. Overall, these findings reinforce the comparable effectiveness of pirfenidone and nintedanib in terms of survival in routine clinical practice.

To further place our findings in a broader real-world context, we compared our functional staging data with large European and international registries, including EMPIRE and INSIGHT-IPF. Comparing our FVC staging data with the EMPIRE registry, we observe that higher FVC values were recorded in Poland, Slovakia, Croatia, and Austria, whereas lower values were recorded in the Czech Republic, Turkey, and Israel [31]. Regarding DLCO staging, our data are consistent with INSIGHT-IPF (DLCO 37.8%) and somewhat lower than the US registry (DLCO 40.6%). We did not establish a correlation between stage and progression for either FVC or DLCO, and thus our study supports the need for other multidimensional risk-prediction models [32].

Different studies have shown that PH is an independent predictor of poor outcomes in patients with IPF [33]. According to screening guidelines for PH, an elevated RVSP is ≥40 mmHg [34]. In our cohort, echocardiographic measurements were elevated in 30 patients. Testing the correlation with progression did not find a statistically significant association; that is, during one-year follow-up, there was no increased incidence of progression in this group of patients.

Meta-analyses of studies conducted in real-world settings have shown that pirfenidone and nintedanib are equally effective at slowing the decline in lung function in patients with IPF. However, the mortality rates and the incidence of acute exacerbations of IPF are higher with both drugs in real-world conditions than those recorded in clinical trials, with patients receiving pirfenidone having shorter survival. The incidence of adverse effects with pirfenidone is lower than with nintedanib, but in both drugs, it is lower than that reported in clinical trials, and no new serious adverse events were reported [35]. Taken together, these minor divergences are likely attributable to real-world heterogeneity, differences in patient selection and follow-up, and unmeasured confounding inherent to retrospective observational studies, rather than true differences in treatment effectiveness.

Only four previous studies have examined the effectiveness of pirfenidone during a follow-up period of more than 2 years in real-world settings [36,37,38,39]. The only available data for nintedanib come from long-term open-label extension studies that followed clinical trials and from real-world data on effectiveness for more than 3 years [40,41,42,43]. Paolo Cameli and colleagues concluded that, in a 10-year follow-up of patients with IPF, antifibrotics are equally effective in terms of mortality and functional disease progression [21].

This study has several limitations. Due to its retrospective design, it is limited by the accuracy and completeness of data recorded in the Heliant system. The relatively small sample size may have limited the ability to detect subtle differences in overall survival between treatment groups. In addition, the non-randomized nature of the study introduces the possibility of residual confounding and treatment selection bias. A substantial proportion of patients were censored during follow-up, particularly at later time points, which may affect the precision of long-term survival estimates. Furthermore, the number of acute exacerbations, hospitalizations, therapy-related adverse events, and treatment adherence was not systematically assessed. Quality-of-life measures and multidimensional prognostic tools (GAP, CPI, PROs) were also not included. Finally, as a single-center real-world study, the generalizability of these findings to other populations and healthcare settings may be limited.

## 5. Conclusions

This retrospective study demonstrated that pirfenidone and nintedanib are equally effective in slowing disease progression in the examined population of IPF patients. However, real-world effectiveness measures often fail to capture patient-relevant outcomes such as quality of life and adverse events. Therefore, the choice between pirfenidone and nintedanib should be individualized, taking into consideration clinical characteristics, comorbidities, concomitant therapies, individual risk of adverse events, and patient preferences. In our cohort, disease progression was strongly associated with a probable UIP pattern on HRCT, supporting current guidelines suggesting that probable UIP has a natural history and prognosis similar to those of definite UIP. In future contexts, patients with a probable UIP pattern should be considered for early recognition, close monitoring, and timely initiation of antifibrotic therapy.

It is essential to emphasize that IPF—a highly lethal disease—is frequently underrecognized. Treatment is often initiated in the later stages, when patients already experience substantial functional impairment and reduced quality of life. Greater focus on screening, prevention, early detection, and early initiation of therapy should become a priority in the coming years, in order to mitigate the devastating consequences of this disease, whose survival rates can, in some cases, be shorter than those observed in oncology patients.

## Figures and Tables

**Table 1:** Baseline characteristics of the examined population. Data are presented as mean ± standard deviation or number, as appropriate. BMI—body mass index; COPD—chronic obstructive pulmonary disease; PH—pulmonary hypertension group 3; HRCT—high-resolution computed tomography; UIP—usual interstitial pneumonia. *p*-values refer to comparisons between treatment groups.

	Population	Nintedanib	Pirfenidone	*p* Value
*n* (%)	76	31 (40.8)	45 (59.2)	-
Male *n* (%)	54	19 (51.3)	35 (77.8)	-
Female *n* (%)	22	12 (38.7)	10 (22.2)	-
Age/years	69.50 ± 8.07	69 ± 6.44	70 ± 9.06	0.531
BMI (kg/m^2^)	26.01 ± 4.65	25.95 ± 5.44	26.6 ± 4.09	0.854
Comorbidities *n* (%)	71 (93.4)	30 (96.8)	41 (91.1)	0.643
Art. hypertension *n* (%)	55 (71)	23 (41.81)	32 (58.19)	0.786
Diabetes *n* (%)	20 (26.31)	10 (50)	10 (50)	0.32
PH *n* (%)	10 (13.15)	3 (30)	7 (70)	0.514
COPD *n* (%)	12 (15.78)	5 (41.66)	7 (58.34)	1.0
Smoking history *n* (%)	66 (73.7%)	22 (71)	34 (75.5)	0.287
Ex-smoker *n* (%)	44 (57.9)	15 (34.09)	29 (65.91)	-
Current smoker *n* (%)	12 (15.8)	7 (58.33)	5 (41.67)	-
Nonsmoker *n* (%)	20 (26.3)	9 (45)	11 (55)	-
HRCT				0.174
Definite UIP *n* (%)	39 (51.3)	13 (33.3)	26 (66.7)	-
Probable UIP *n* (%)	37 (48.7)	18 (48.6)	19 (51.4)	-
Progression *n* (%)	35 (46.1)	18 (58.06)	17 (37.77)	0.81

**Table 2:** Pulmonary function over time and treatment (mean ± SD); FVC—Forced vital capacity; DLCO—Diffusing capacity of the lungs for carbon monoxide, *p*-values showing no difference between groups in FVC and DLCO decline.

	Baseline	6 Months	One Year	*p* Value
Nintedanib FVC %	79.16 ± 22.660	79.39 ± 23.617	74.97 ± 22.60	*p* = 0.383
Pirfenidone FVC %	79.31 ± 15.98	75.78 ± 15.26	78.29 ± 17.53	-
Nintedanib DLCO %	38.54 ± 14.68	33.21 ± 5.58	32.27 ± 14.32	*p* = 0.667
Pirfenidone DLCO %	36.04 ± 11.39	32.82 ± 10.88	32.47 ± 11.61	-

**Table 3:** Distance passed over time in relation to treatment (mean ± SD). 6MWTD—6-min walk test distance, *p*-value showing no difference between groups in 6MWTD decline.

6MWTD	Baseline	6 Months	One Year	*p* Value
Nintedanib (m)	341.44 ± 94.32	322.03 ± 117.31	319.96 ± 131.69	*p* = 0.566
Pirfenidone (m)	335.14 ± 100	317.71 ± 116.89	294.17 ± 133.18	-

**Table 4:** Frequency of DLCO and FVC stages according to treatment.

Stage DLCO	I (>55%)	II (35–55%)	III (<35%)
Nintedanib (*n*)	1	13	17
Pirfenidone (*n*)	3	11	31
Stage FVC	I (>80%)	II (50–79%)	III (<50%)
Nintedanib (*n*)	17	13	1
Pirfenidone (*n*)	23	22	0

## Data Availability

The original contributions presented in this study are included in the article. Further inquiries can be directed to the corresponding author.

## Abbreviations

The following abbreviations are used in this manuscript:

IPF	Idiopathic pulmonary fibrosis
FVC	Forced vital capacity
DLCO	Diffusion capacity for carbon monoxide
6MWT	Six-minute walk distance test
HRCT	High-resolution computed tomography
UIP	Usual interstitial pneumonia
TGF-β	Transforming growth factor-β
PDGF	Platelet-derived growth factor
FGF	Fibroblast growth factor
VEGF	Vascular endothelial growth factor
BMI	Body mass index
COPD	Chronic obstructive pulmonary disease
HTN	Arterial hypertension
DM	Diabetes mellitus
PH	Pulmonary arterial hypertension group 3
EF	Ejection fraction
ECHO	Ultrasound of the heart
RVSP	Systolic pressure of the right ventricle
GERD	Gastroesophageal reflux disease

## References

[1] Maher T.M., Bendstrup E., Dron L., Langley J., Smith G., Khalid J.M., Patel H., Kreuter M. (2021). Global incidence and prevalence of idiopathic pulmonary fibrosis. Respir. Res..

[2] Richeldi L., Collard H.R., Jones M.G. (2017). Idiopathic pulmonary fibrosis. Lancet.

[3] Rajesh R., Atallah R., Bärnthaler T. (2023). Dysregulation of metabolic pathways in pulmonary fibrosis. Pharmacol. Ther..

[4] Chianese M., Screm G., Salton F., Confalonieri P., Trotta L., Barbieri M., Ruggero L., Mari M., Reccardini N., Geri P. (2024). Pirfenidone and Nintedanib in Pulmonary Fibrosis: Lights and Shadows. Pharmaceuticals.

[5] Provenzani A., Vinci D.L., Alaimo M., Di Maria S., Tuzzolino F., Floridia G., Di Stefano R., Carollo A., Callari A., Polidori P. (2025). Real-world insights into safety, tolerability, and predictive factors of adverse drug reactions in treating idiopathic pulmonary fibrosis with pirfenidone and nintedanib. Ther. Adv. Drug Saf..

[6] Raghu G., Remy-Jardin M., Richeldi L., Thomson C.C., Inoue Y., Johkoh T., Kreuter M., Lynch D.A., Maher T.M., Martinez F.J. (2022). Idiopathic Pulmonary Fibrosis (an Update) and Progressive Pulmonary Fibrosis in Adults: An Official ATS/ERS/JRS/ALAT Clinical Practice Guideline. Am. J. Respir. Crit. Care Med..

[7] Graham B.L., Brusasco V., Burgos F., Cooper B.G., Jensen R., Kendrick A., MacIntyre N.R., Thompson B.R., Wanger J. (2017). 2017 ERS/ATS standards for single-breath carbon monoxide uptake. Eur. Respir. J..

[8] Graham B.L., Steenbruggen I., Miller M.R., Barjaktarevic I.Z., Cooper B.G., Hall G.L., Hallstrand T.S., Kaminsky D.A., McCarthy K., McCormack M.C. (2019). Standardization of Spirometry—2019 Update. Am. J. Respir. Crit. Care Med..

[9] ATS Committee (2002). ATS Statement: Guidelines for the Six-Minute Walk Test. Am. J. Respir. Crit. Care Med..

[10] Kolb M., Collard H.R. (2014). Staging of idiopathic pulmonary fibrosis: Past, present and future. Eur. Respir. Rev..

[11] Arshad H.M.E., Ali F., Babar A., Raza M.Z., Maqsood M., Ameer A. (2025). Survival impact and safety comparison of pirfenidone and nintedanib for idiopathic pulmonary fibrosis: A meta-analysis. Sarcoidosis Vasc. Diffuse Lung Dis..

[12] Hadda V., Guleria R. (2020). Antifibrotic drugs for idiopathic pulmonary fibrosis: What we should know?. Indian J. Med. Res..

[13] Moor C.C., Mostard R.L.M., Grutters J.C., Bresser P., Aerts J.G.J.V., Dirksen C.D., Kimman M.L., Wijsenbeek M.S. (2020). Patient expectations, experiences and satisfaction with nintedanib and pirfenidone in idiopathic pulmonary fibrosis: A quantitative study. Respir. Res..

[14] Neely M.L., Hellkamp A.S., Bender S., Todd J.L., Liesching T., Luckhardt T.L., Oldham J.M., Raj R., White E.S., Palmer S.M. (2023). Lung function trajectories in IPF. Respir. Res..

[15] King T.E., Bradford W.Z., Castro-Bernardini S., Fagan E.A., Glaspole I., Glassberg M.K., Gorina E., Hopkins P.M., Kardatzke D., Lancaster L. (2014). A phase 3 trial of pirfenidone in patients with idiopathic pulmonary fibrosis. N. Engl. J. Med..

[16] Richeldi L., Du Bois R.M., Raghu G., Azuma A., Brown K.K., Costabel U., Cottin V., Flaherty K.R., Hansell D.M., Inoue Y. (2014). Efficacy and safety of nintedanib in idiopathic pulmonary fibrosis. N. Engl. J. Med..

[17] Schmidt S.L., Tayob N., Han M.K., Zappala C., Kervitsky D., Murray S.K., Wells A.U., Brown K.K., Martínez F.J., Flaherty K.R. (2014). Predicting IPF disease course from past pulmonary function trends. Chest.

[18] du Bois R.M., Weycker D., Albera C., Bradford W.Z., Costabel U., Kartashov A., King T.E., Lancaster L., Noble P.W., Sahn S.A. (2011). Forced vital capacity in patients with idiopathic pulmonary fibrosis: Test properties and minimal clinically important difference. Am. J. Respir. Crit. Care Med..

[19] Latsi P.I., du Bois R.M., Nicholson A.G., Colby T.V., Bisirtzoglou D., Nikolakopoulou A., Veeraraghavan S., Hansell D.M., Wells A.U. (2003). Fibrotic idiopathic interstitial pneumonia: Prognostic value of longitudinal functional trends. Am. J. Respir. Crit. Care Med..

[20] Plantier L., Cazes A., Dinh-Xuan A.T., Bancal C., Marchand-Adam S., Crestani B. (2018). Physiology of the lung in idiopathic pulmonary fibrosis. Eur. Respir. Rev..

[21] Cameli P., Refini R.M., Bergantini L., d’Alessandro M., Alonzi V., Magnoni C., Rottoli P., Sestini P., Bargagli E. (2020). Long-term follow-up of patients with idiopathic pulmonary fibrosis treated with pirfenidone or nintedanib: A real-life comparison study. Front. Mol. Biosci..

[22] Singh S.J., Puhan M.A., Andrianopoulos V., Hernandes N.A., Mitchell K.E., Hill C.J., Lee A.L., Camillo C.A., Troosters T., Spruit M.A. (2014). Measurement properties of walking tests in chronic respiratory disease. Eur. Respir. J..

[23] Nathan S.D., du Bois R.M., Albera C., Bradford W.Z., Costabel U., Kartashov A., Noble P.W., Sahn S.A., Valeyre D., Weycker D. (2015). Validation of test performance characteristics and minimal clinically important difference of the 6-minute walk test in patients with idiopathic pulmonary fibrosis. Respir. Med..

[24] Lederer D.J., Arcasoy S.M., Wilt J.S., D’Ovidio F., Sonett J.R., Kawut S.M. (2006). Six-Minute-Walk Distance Predicts Waiting List Survival in Idiopathic Pulmonary Fibrosis. Am. J. Respir. Crit. Care Med..

[25] Lee J.H., Park H.J., Kim S., Kim Y.-J., Kim H.C. (2023). Epidemiology and comorbidities in IPF: Nationwide cohort. BMC Pulm. Med..

[26] Brown S.W., Dobelle M., Padilla M., Agovino M., Wisnivesky J.P., Hashim D., Boffetta P. (2019). IPF and lung cancer: Systematic review and meta-analysis. Ann. Am. Thorac. Soc..

[27] Chen T., Yin C.-S., Wang P., Li Q.-H., Shao C., Huang H., Song L., Zhang W.-H., Xu Z.-J. (2024). Differences Between Patients with Probable UIP and Definite UIP on HRCT in Idiopathic Pulmonary Fibrosis: A Real-World Cohort Study. J. Clin. Med..

[28] Raghu G., Chen S.Y., Yeh W.S., Maroni B., Li Q., Lee Y.-C., Collard H.R. (2014). Idiopathic pulmonary fibrosis in US Medicare beneficiaries aged 65 years and older: Incidence, prevalence, and survival, 2001–11. Lancet Respir. Med..

[29] Hopkins R.B., Burke N., Fell C., Dion G., Kolb M. (2016). Epidemiology and survival of idiopathic pulmonary fibrosis from national data in Canada. Eur. Respir. J..

[30] Salciccioli J.D., Marshall D.C., Goodall R., Crowley C., Shalhoub J., Patel P., Molyneaux P.L. (2022). Interstitial lung disease incidence and mortality in the UK and the European Union: An observational study, 2001–2017. ERJ Open Res..

[31] Kolonics-Farkas A.M., Šterclová M., Mogulkoc N., Lewandowska K., Müller V., Hájková M., Kramer M., Jovanovic D., Tekavec-Trkanjec J., Studnicka M. (2021). Differences in Baseline Characteristics and Access to Treatment of Newly Diagnosed Patients with IPF in the EMPIRE Countries. Front. Med..

[32] Ley B., Ryerson C.J., Vittinghoff E., Ryu J.H., Tomassetti S., Lee J.S., Poletti V., Buccioli M., Elicker B.M., Jones K.D. (2012). A multidimensional index and staging system for idiopathic pulmonary fibrosis. Ann. Intern. Med..

[33] Kimura M., Taniguchi H., Kondoh Y., Kimura T., Kataoka K., Nishiyama O., Aso H., Sakamoto K., Hasegawa Y. (2013). Pulmonary hypertension as a prognostic indicator at the initial evaluation in idiopathic pulmonary fibrosis. Respiration.

[34] Augustine D.X., Coates-Bradshaw L.D., Willis J., Harkness A., Ring L., Grapsa J., Coghlan G., Kaye N., Oxborough D., Robinson S. (2018). Echocardiographic assessment of pulmonary hypertension: A guideline protocol from the British Society of Echocardiography. Echo Res Pract..

[35] Kou M., Jiao Y., Li Z., Wei B., Li Y., Cai Y., Wei W. (2024). Real-world safety and effectiveness of pirfenidone and nintedanib in the treatment of idiopathic pulmonary fibrosis: A systematic review and meta-analysis. Eur. J. Clin. Pharmacol..

[36] Bando M., Yamauchi H., Ogura T., Taniguchi H., Watanabe K., Azuma A., Homma S., Sugiyama Y., The Japan Pirfenidone Clinical Study Group (2016). Clinical experience of the long-term use of pirfenidone for idiopathic pulmonary fibrosis. Intern. Med..

[37] Tzouvelekis A., Karampitsakos T., Ntolios P., Tzilas V., Bouros E., Markozannes E., Malliou I., Anagnostopoulos A., Granitsas A., Steiropoulos P. (2017). Longitudinal ‘real-world’ outcomes of pirfenidone in idiopathic pulmonary fibrosis in Greece. Front. Med..

[38] Section I., Zurkova M., Registry I., Kriegova E., Kolek V., Lostakova V., Sterclova M., Bartos V., Doubkova M., Binkova I. (2019). Effect of pirfenidone on lung function decline and survival: 5-yr experience from a real-life IPF cohort from the Czech EMPIRE registry. Respir. Res..

[39] Vietri L., Cameli P., Perruzza M., Cekorja B., Bergantini L., D’aLessandro M., Refini R.M., Pieroni M., Fossi A., Bennett D. (2020). Pirfenidone in idiopathic pulmonary fibrosis: Real-life experience in a referral centre. Ther. Adv. Respir. Dis..

[40] Richeldi L., Kreuter M., Selman M., Crestani B., Kirsten A.-M., Wuyts W.A., Xu Z., Bernois K., Stowasser S., Quaresma M. (2018). Long-term treatment of patients with idiopathic pulmonary fibrosis with nintedanib: Results from the TOMORROW trial and its open-label extension. Thorax.

[41] Crestani B., Huggins J.T., Kaye M., Costabel U., Glaspole I., Ogura T., Song J.W., Stansen W., Quaresma M., Stowasser S. (2019). Long-term safety and tolerability of nintedanib in patients with idiopathic pulmonary fibrosis: Results from the open-label extension study, INPULSIS-ON. Lancet Respir. Med..

[42] Antoniou K., Markopoulou K., Tzouvelekis A., Trachalaki A., Vasarmidi E., Organtzis J., Tzilas V., Bouros E., Kounti G., Rampiadou C. (2020). Efficacy and safety of nintedanib in a Greek multicentre idiopathic pulmonary fibrosis registry: A retrospective, observational, cohort study. ERJ Open Res..

[43] Song J.W., Ogura T., Inoue Y., Xu Z., Quaresma M., Stowasser S., Stansen W., Crestani B. (2020). Long-term treatment with nintedanib in Asian patients with idiopathic pulmonary fibrosis: Results from INPULSIS R©-ON. Respirology.

